# Evaluating the Influences of Natural Resources and Ageing People on CO_2_ Emissions in G-11 Nations: Application of CS-ARDL Approach

**DOI:** 10.3390/ijerph19031449

**Published:** 2022-01-27

**Authors:** Usman Mehmood, Ephraim Bonah Agyekum, Solomon Eghosa Uhunamure, Karabo Shale, Ayesha Mariam

**Affiliations:** 1 Department of Political Science, University of Management and Technology, Lahore 54770, Pakistan; 2 Remote Sensing, GIS and Climatic Research Lab (National Center of GIS and Space Applications), Centre for Remote Sensing, University of the Punjab, Lahore 54590, Pakistan; Ayeshamariam60@gmail.com; 3 Department of Nuclear and Renewable Energy, Ural Federal University Named after the First President of Russia Boris, 19 Mira Street, 620002 Ekaterinburg, Russia; agyekumephraim@yahoo.com; 4 Faculty of Applied Sciences, Cape Peninsula University of Technology, P.O. Box 652, Cape Town 8000, South Africa; uhunamures@cput.ac.za (S.E.U.); shalek@cput.ac.za (K.S.)

**Keywords:** G-11 countries, ageing population, natural resources, globalization, CS-ARDL

## Abstract

Globalization as well as the ratio of ageing people in the group of 11 (G-11) countries has seen a rapid increase in recent years. Therefore, this study aims to provide effective policy recommendations for sustainable development goals 13, 8, and 7, for the G-11 countries. This work estimates the impact of natural resources and the ageing population on the emission of carbon dioxide (CO_2_) in G-11 countries using panel data from 1990–2020. For empirical results, second-generation methods were applied. The Westerlund co-integration test that assesses co-integration confirms the firm association among the parameters, and the values of coefficient of the cross-sectional autoregressive distributed lag (CS-ARDL) approach show that a 1% increase in the ageing population will lower the emissions of CO_2_ by 13.41% among G-11 countries. Moreover, the findings show that there exists an environmental Kuznets curve (EKC) among natural resources, globalization, economic growth, ageing people, and the emission of CO_2_. Based on the findings, this work presents some important policy implications for achieving sustainable growth in the G-11 countries. These countries need to lower the amount of energy obtained from fossil fuels to improve air quality.

## 1. Introduction

Rapid industrialization has created hurdles on the way to achieving sustainable development. According to United Nations (UN), developed and developing nations are striving to address climate problems but industrialization is making their efforts fruitless. To accomplish the economic targets of various countries, different resources are being shared across borders. These trade activities have been possible through globalization. Globalization affects the process of production, which ultimately affects environmental quality [1,2,3,4]. 

Today, different economies are creating economic targets by enhancing cross border trade. Most countries, however, overlooked the factors that could affect environmental quality when pursuing their economic goals [4]. The group of 11 (G-11) countries was formed on 20 September 2006, when most of the countries were in their developing stages. This group was established to enhance their economic progress by cooperation. Since its formation, the G-11 countries have shown remarkable economic progress [5]. After joining the Paris agreement, the (G-11) nations have shown a strong commitment to reduce environmental pollution and they are revising their current economic and demographic policies. This includes environmental actions, usage of clean energy, and improved living standards. 

Due to improving health facilities, the ageing population is increasing and the rise in the ageing population may have environmental consequences. According to the World Bank, the ageing population in G-11 countries has seen a rapid increase. This population is projected to reach 923 million by 2050 [6], and the governments of these countries are not well prepared for this demographic change. In an economic context, a labour supply shortage might be created by an upsurge in ageing people [7]. However, in the context of environmental quality, ecological degradation is caused by moving ageing people, household pattern variations, and building of separate homes for such people. Ageing people have environmental awareness but their preferences for a cleaner environment may vary. Ageing people have less preference for the use of private vehicles to move from one place to another. At the same time, they may need additional energy in terms of health-related facilities and if the energy is coming from non-renewable energy resources, it will degrade the environment. Therefore, it is essential to probe the linkages of ageing people with emissions of CO_2_. 

The objective of the current study is to evaluate the influence of the ageing population on CO_2_ emissions in G-11 nations. Furthermore, considering the importance of other socio-economic variables, this work includes energy use, globalization, economic growth, energy innovations, and natural resources on CO_2_ emissions. To the best of the author’s knowledge, there is a gap in literature, and very few studies have investigated the factors of environmental degradation in the context of SDGs for G-11 countries. Also, for the analytical framework, the environmental Kuznets curve (EKC) has not been addressed. EKC proposes that after reaching a threshold level, economic activity may reduce environmental pollution. This may be due to environmental awareness or the use of efficient means of energy. This work probes the EKC among globalization, natural resources, economic growth, and ageing populations in G-11 nations. 

For effective policy instruments, this work utilizes the second-generation methods because the first-generation methods may not incorporate the cross-sectional heterogeneities, which the study seeks to address. Therefore, the present study uses the cross-sectional autoregressive distributed lag (CS-ARDL) technique. This method controls the structural similarities to provide the effects of independent parameters on the dependent parameters. Considering these advantages, this work uses the CS-ARDL instead of the traditional autoregressive distributed lag (ARDL) [8]. 

To capture the evolutionary impacts on CO_2_ emissions, a suitable theoretical model is required. Hence, the EKC by Grossman and Krueger [9] has been employed to determine the evolutionary associations among the variables. The paper is structured as follows: a literature background is described in Section 2, the methodology and data are described in Section 3, the fourth section comprises an analysis of the results, and the last section outlines the conclusions of the study. 

## 2. Literature Review

### 2.1. Energy and Air Pollution

The EKC hypothesis has been discussed widely by environmental economists [9,10]. This assumption presented that economic growth influences environmental pollution in three ways: technique, composition, and scale [11,12]. Currently, several studies have posited that innovations in energy are the key factors that lower global warming [7,13]. According to Torras et al. [14], technical novelties lower environmental pollution but recent literature also suggests that the scale effect can be lowered by using low carbon emissions technologies. 

### 2.2. Natural Resources and Air Pollution

Several studies have shown that more natural resources are important to impact economic growth. For example, Auty [15] found that rich natural resources slow down the pace of economic growth. However, Bravo-Ortega and de Gregorio [16], observed that natural resources increase income but have a negative effect on the national growth rate. Shahbaz et al. [17], validated the natural resource curse hypothesis. Brunnschweiler and Bulte [18], described the difference between natural resource dependence and abundance. They presented that natural resource abundance increases economic growth whereas gross domestic product (GDP) is unaffected by natural resource dependence. Balsalobre-Lorente et al. [7], argued that the abundance of natural resources reduces CO_2_ emissions in European countries. They argued that countries with ample natural resources utilize them instead of fossil fuels and maintain economic growth. However, Danish et al. [19], presented contradictory evidence for Brazil, Russia, India, China and South Africa (the BRICS nations).

### 2.3. Globalization and Air Pollution

Globalization is increasing political, social, and economic integration across the globe. Dreher [20], reported that globalization put a positive impact on the growth of an economy. Dollar and Kraay [21] have observed the positive impact of globalization on economic growth. Similarly, Alam [22] found a positive nexus between environmental degradation and globalization. Kahuthu [23], investigated the association between CO_2_ emissions and economic growth. They observed that globalization is playing a moderating role in this association by importing efficient technologies. Globalization is increasing GDP but lowering CO_2_ emissions. Shahbaz et al. [24], found that globalization has degraded the environment. Shahbaz et al. [11], investigated the emissions of CO_2_–globalization nexus in Indian economic growth. They also found that globalization is degrading the environment. Shahbaz et al. [25] suggested that globalization is triggering foreign direct investment, which enhances the reckless use of non-renewable energy, which contaminates the quality of the environment. However, for the Australian economy Shahbaz et al. [26], found that globalization is environmentally friendly. They argued that due to effective resource policy and administrative grip, globalization is a blessing to Australia.

### 2.4. Ageing and Air Pollution

Some studies have shown the association between ageing and air pollution [27]. York et al. [28] and Shi et al. [29] found that an ageing population can create more emissions of CO_2_. Fan et al. [30] argued that the working class is lowering air quality in developing countries, but this class is improving air quality in developed nations. However, reference [31] argued that the elderly people use fewer resources and prefer public transportation and therefore they are environmentally friendly. Hassan and Salim [32], found that aged people are reducing emissions of CO_2_ by 1.55%. O’Neill et al. [33], found that aged people do not participate in labour activities, and they slow economic growth with little to no emissions of CO_2_.

Contrary to this, various studies have shown the adverse impacts of ageing people on the emission of CO_2_. Farzin et al. [27] indicated that a society with more aged people will generate more CO_2_ emissions. Menz and Welsch [34], presented that aged people use up more energy and increase CO_2_ emissions. Menz et al. [35] found that the ageing people–CO_2_ emissions linkage depends upon the country’s position relative to development. Thalmann [36], presented that the wish for a cleaner environment decreases with age. They further highlighted that although elderly people are affected by environmental changes, they are not going to obtain the benefits of environmental regulations in the future. This thought further diminishes environmental awareness. According to Liddle and Lung [37], middle-age people require less energy requirements, but at an early and elderly age, they require more energy. Liddle [38], found the U-shaped linkages between ageing people and domestic energy consumption. It was observed that the youngest and elderly people positively affect energy demand. 

Considering the potential impact of ageing people on the environment, this work attempts to enhance the current wave of knowledge. 

### 2.5. Research Gap

The aforementioned literature above shows that different studies have contradictory results about the factors of environmental degradation. The contradictory results may be due to the level of development and the sample of variables collected from the countries. To the best of the authors’ knowledge, there is a gap in literature, and very few studies have investigated the factors of environmental degradation in the context of SDGs for G-11 countries. Moreover, for the analytical framework, the EKC hypothesis has not been addressed. This gap in the literature is addressed by incorporating ageing people. 

## 3. Data and Empirical Estimation

**Figure 1:**
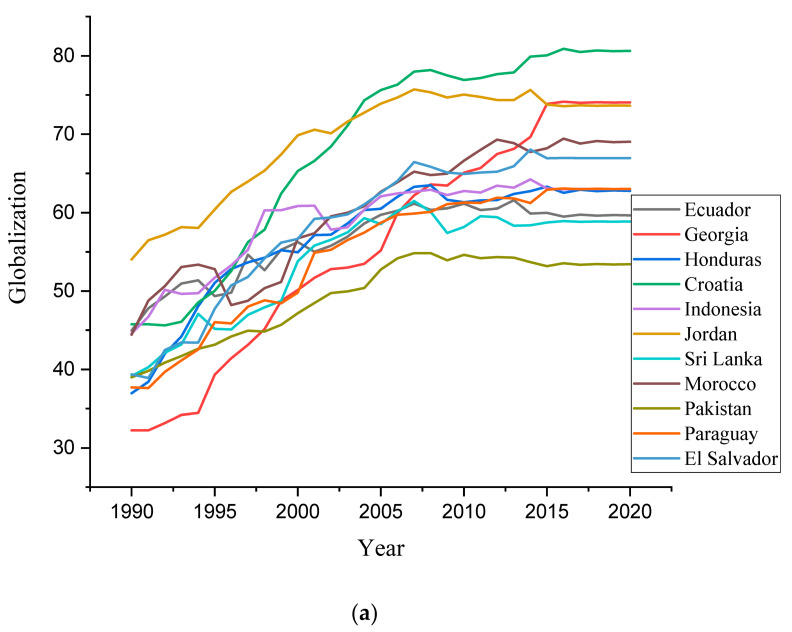

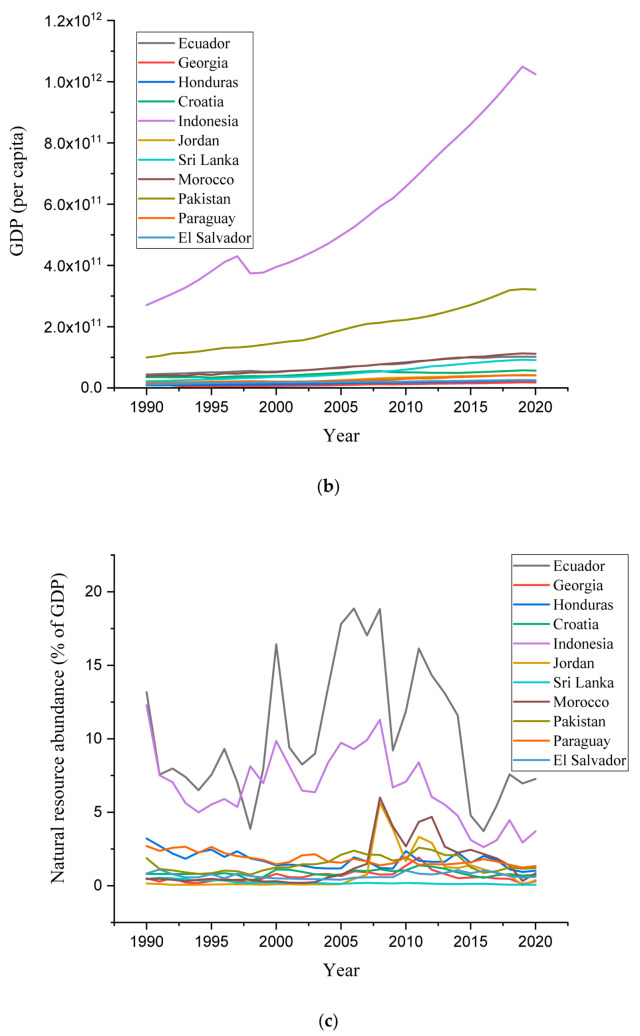

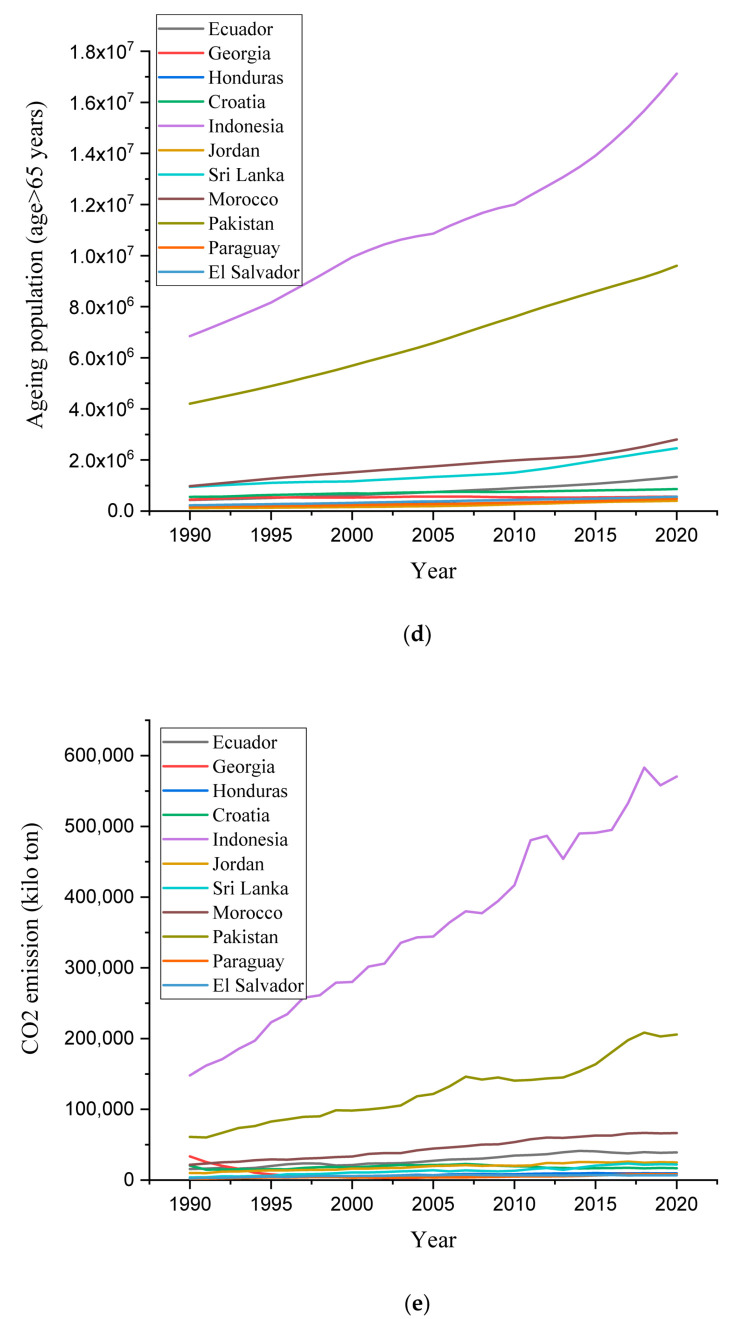
Graphical representation of variables used for the study for the various countries (**a**) globalization, (**b**) GDP per capita, (**c**) natural resource abundance, (**d**) ageing population, (**e**) CO_2_ emissions.

This work attempts to probe the impact of ageing people on CO_2_ emissions by controlling the other socio-economic factors of globalization, natural resources, GDP, and energy use. In doing so, this work utilizes the annual data of 1990–2020 for G-11 nations of Croatia, Jordan, Ecuador, El Salvador, Georgia, Honduras, Morocco, Indonesia, Paraguay, Pakistan, and Sri Lanka. The data for GDP per capita (constant terms), CO_2_ emissions (kilo tons), natural resources (% of GDP), energy use (kg of oil equivalent per capita), research, and development (number of patents), and the ageing population 65 and above were used. All the data were obtained from the World Bank [39] except the data for globalization, which was obtained from the KOF Economic Institute [40]. Figure 1a–e illustrates an increasing trend in globalization, GDP per capita, natural resource abundance, research and development, ageing population, and CO_2_ emissions in G-11 countries. Croatia showed the highest globalization during 1990 to 2020. Similarly, Indonesia had the highest economic growth among the G-11 countries during the study period. The highest natural resource was in Ecuador. Indonesia had the highest number of ageing people during the study period.

Before the econometric analysis, all data were transformed into their natural logarithms. This form eliminates the problems of multicollinearity and provides robust findings [41,42,43]. This work follows the study of Balsalobre-Lorente et al. [44] in applying the empirical model, which is as follows:

(1) $$\begin{array}{cc} {\mathrm{lnCO}}_{2t\;}={\;\beta }_{0\;}+ & {\;\beta }_{1\;}{\mathrm{lnGL}}_{t\;}+\beta_{2\;}{\mathrm{lnGl}}_{t}^{2}+\beta_{3\;}{\mathrm{lnGDP}}_{t\;}+{\;\beta }_{4\;}{\mathrm{lnGDP}}_{t}^{2}+{\;\beta }_{5\;}{\mathrm{lnNAT}}_{t\;}+{\;\beta }_{6\;}{\mathrm{lnNAT}}_{t}^{2}+{\;\beta }_{7\;}{\mathrm{lnAG}}_{t\;}\\ & +\beta_{8\;}{\mathrm{lnAG}}_{t}^{2}+\beta_{9\;}\mathrm{lnEN}+{\;\beta }_{10\;}{\mathrm{lnRD}}_{t\;}+{\;є}_{t\;} \end{array}$$

where, CO_2_, GL, GDP, NAT, AG, EN, and RD represent the CO_2_ emissions (kilo ton), the overall index of globalization, GDP per capita (constant term), natural resource abundance (% of GDP), ageing population 65 years and above, energy use (kg of oil equivalent per capita), and research and development (number of patents).

Table 1 presents the descriptive statistics of the variables, which shows that GDP, globalization, and number of ageing people in the population have the highest values. 

Table 2 shows the description and sources of data. For econometric analysis, this work adopts the second-generation methodology. There is a reason to use second-generation methods because the datasets obtained for South Asian countries may suffer from cross-section dependence (CD) due to common traditional methods, social norms, and economic policies. It might not be able to provide robust results. A second-generation unit root test is applied to find the order of integration among the panel data. The CS-generation technique is applied to present the values of long- and short-run coefficients. 

### Cross-Sectional Autoregressive Distributed Lag (CS-ARDL)

This work seeks to probe the linkages between economic growth, natural resources, energy use, globalization, ageing people, and CO_2_ emissions for a panel of G-11 nations. Panel estimations can generate unreliable results because of the existence of cross-section dependence and slope heterogeneity issues. These issues are not considered by the traditional estimation techniques of FMOL and DOLS [45]. The issue of slope heterogeneity and CD is efficiently handled by the CS-ARDL approach, which is not catered for by the FMOL and DOLS techniques. Therefore, the current study used the CS-ARDL method to calculate the values of long- and short-run coefficients. This method caters for heterogeneity and CD problems by applying dynamic common correlated impact predictors [46,47]. Equation (1) represents the mathematical form of the CS-ARDL:

(2) $$H_{i,t}=\overset{p_{w}}{\underset{I=0}{\sum }}\gamma_{I,i}W_{i,t-1}+\overset{p_{z}}{\underset{I=0}{\sum }}\beta_{I,i}Z_{i,t-I}+\varepsilon_{i,t}$$

Equation (1) represents the ARDL model; if we use Equation (5), by taking CD, it will produce uncertain results. Equation (4) is revised using averages of CS one by one repressor parameters. This will permit us to remove inappropriate interpretations concerning the existence of the threshold effect generated by CD [8].

(3) $$H_{it}=\overset{a_{w}}{\underset{I=0}{\sum }}\gamma_{I,i},H_{i,t-1}+\overset{a_{z}}{\underset{I=0}{\sum }}\beta_{I,i}Z_{i.t-I}+\overset{a_{x}}{\underset{I=0}{\sum }}{\alpha^{\prime }}_{i},I{\bar{X}}_{t-I}+\varepsilon_{i,t}$$

where the average value of dependent and independent parameters can be calculated by using the following equation:

$${\bar{X}}_{t-I}={\bar{H}}_{i,t-I}{\bar{Z}}_{i,t-I}$$

Existing lags among all the variables are denoted by *a_w_*, *a_x_* and *a_z_*. $H_{it}$ denotes emission of carbon per capita depending upon its utilization and $Z_{i.t}$ represents all the independent variables. Furthermore, *X* denote the average of CS (disregarding the trends) to overwhelm the spillover issues [48]. The CS-ARDL method estimates the long-run coefficients by using short-run coefficients as its input. Equations (4)–(6) represent the mean group (MG) predictor and the value of long-run and short-run coefficients, respectively:

(4) $${\hat{\varphi }}_{MG}=\frac{1}{N}\overset{N}{\underset{i=1}{\sum }}{\hat{\varphi }}_{i}$$

(5) $${\hat{\varphi }}_{CS-ARDL,i}=\frac{\sum_{I=0}^{p_{z}}\beta_{I,i}^{pw}}{1-\Sigma_{I=0}}\hat{\gamma_{I,i}}$$

(6) $$\Delta H_{i,t}=\vartheta_{i}\left[H_{i,t-1}-\varphi_{i}Z_{i,t}\right]-\overset{a_{w-1}}{\underset{I=1}{\sum }}\gamma_{I,i}\Delta_{I}H_{i,t-1}+\overset{a_{ws-1}}{\underset{I=1}{\sum }}\beta_{I,i}\Delta_{I}Z_{i,t}+\overset{a_{x}}{\underset{I=0}{\sum }}{\alpha^{\prime }}_{i},I{\bar{X}}_{t-I}+\varepsilon_{i,t}$$

where $\Delta_{I}=t-\left(t-1\right)$,

(7) $$\hat{\delta_{i}}=-(1-\overset{a_{w}}{\underset{I=1}{\sum }}{\hat{\gamma }}_{I,t})$$

(8) $$\varphi_{i}=\frac{\sum_{I=0}^{a_{z}}\beta_{I,i}^{aw}}{\hat{\delta_{i}}}$$

(9) $${\hat{\bar{\varphi }}}_{\mathrm{MG}}\;=\frac{1}{N}\sum_{i=1}^{N}\hat{\varphi }$$

In the CS-ARDL approach, the economy achieves an equilibrium state as soon as the value of the error correction mechanism (ECM) approaches −1.

## 4. Results and Discussion

For panel data analysis, it is important to be sure of the CD. For this purpose, Table 3 shows the results that reject the null hypothesis of CD among the selected variables, i.e., CO_2_, GL, GDP, AG, NAT, and RD, which confirms that the entire data have a CD at a 1% level. Thus, the results imply that a shock in one country will spill over to the other countries as well. These empirical findings agree with that of Mehmood et al. [49] and Musah at al. [50].

Before the application of long-run analysis, it is required to know the integration order of the data. Therefore, this work applies the CIPS unit root test. Table 4 indicates the results of the unit root test which reveals that the panel data are integrated at the first difference. The results shows that in the CIPS unit root test almost all variables are integrated at the first difference except NAT, which is also integrated at this level. This indicates that except NAT, all remaining variables of interest acquired stationarity after the first difference indicating the integration sequence among the data. The findings are supported by the following studies of Musah et al. [50] and Adamu et al. [51].

After the CD and unit root test, the Hashem Pesaran and Yamagata [52] test was incorporated. A slope heterogeneity test was done to examine the slope heterogeneity between the selected variables. Table 5 depicts the analysis of heterogeneity of slope as measured by Pesaran and Yamagata [52]. This test was used to assess the coefficients of heterogeneous and homogenous slopes from the study. This test confirms the heterogeneity at the 1% significance level.

The findings of Westerlund and Edgerton [53] are presented in Table 6, which depicts the null hypotheses of no co-integration between the parameters in the existence of serial correlation, CD, and heterogeneity. The findings reject the null hypotheses with no mean shift and regime shift. This verifies the existence of a co-integrating association among the CO_2_, GL, GDP, NAT, AG, EN, and RD at a 1% significance level. The results are consistent with Menz and Welsch [34].

Table 7 shows the findings of the CS-ARDL, which shows different insights. The findings are presented sequentially considering the influence of globalization on the emissions of CO_2_. In both short- and long-run estimations, the coefficient of globalization is positive and statistically significant. It is evident that globalization is exerting a positive impact on CO_2_ emissions, but the square of globalization is negatively correlated with CO_2_ emissions. This means that the evolutionary impact of globalization is inverted in the U-shape of CO_2_ emissions in G-11 countries. Globalization increases economic opportunities and also makes room for importing efficient technologies to produce clean energy. This result is similar to the results of Sinha et al. [54]. The coefficient of GDP is positive having a value of 9.75% at a 5% significance level, whereas the square of the GDP is negatively associated with environmental degradation. This implies that the evolutionary impact of economic growth is also inverted in the U-shape, which means that recent economic growth is contaminating the environment. In the future, economic growth will improve air quality. Empirical findings by Balsalobre-Lorente et al. [7], Mehmood and Tariq [55] and Qayyum et al. [56] align with the findings of the study. The finding from the study also indicates that G-11 countries are spending on non-renewable resources in the energy sector but in the future, the ratio of renewable energy to the final energy output will increase which will lead to an improvement in air quality. The findings align with that of Mehmood et al. [55] and Abid et al. [57].

The evolutionary effect of natural resources on the emissions of CO_2_ is an inverted U-shape. This means that dependence on natural resources is lowering air quality, but less consumption of natural resources is improving air quality in G-11 countries. The influence of natural resources on the emissions of CO_2_ can be better explained if the joint effects of innovations in the energy sector and the use of energy on the emissions of CO_2_ are examined. It can be observed that the impact of research and development on the emissions of CO_2_ are negative. This means that research and development are lowering CO_2_ emissions in G-11 countries. 

Lastly, the effect of the ageing population on CO_2_ emissions shows that, currently, elderly people positively improve the air quality but in the future this association becomes inverse. This outcome rejects the existence of EKC between ageing population and CO_2_ emissions. This confirms that the current changing demographic patterns in the G-11 countries are environmentally friendly. However, in the future, due to the ageing population growth, they will negatively affect air quality. This outcome is similar to the findings of Hamza et al. [58].

## 5. Conclusions

During the last few years, the G-11 countries have made commitments to lower the concentration of CO_2_ emissions and to improve air quality. These commitments require a comprehensive environmental policy. Therefore, considering the importance of SDGs in the G-11 countries, this work incorporates globalization and the ageing population to present some important recommendations. This work will be helpful for policymakers to achieve SDGs 13, 8, and 7. This study has proposed a comprehensive policy recommendation by analyzing the role of globalization, research and development, and ageing people. The study shows that the G-11 countries are spending on non-renewable resources in the energy sector but, in the future, the ratio of renewable energy to the final energy output will increase which will lead to an improvement in air quality. This work also revealed the need for policymakers to improve the ratio of renewable energy to the final energy output utilized in the industrial sectors. In increasing the ratio of renewable energy, the governments of these countries need to give special attention to employment opportunities because this aspect can be a hurdle in achieving sustainable development. The impacts of research and development on the emissions of CO_2_ are negative. This means that research and development are lowering CO_2_ emissions in the G-11 countries. Research and development will help invent renewable energy technologies. Currently, ageing people are environmentally friendly but in the future ageing people will start to contaminate air quality by increasing the CO_2_ emissions. This result is important for policymakers, and they should divert their attention towards the environmental awareness of ageing people. 

The role of natural resources is very important for achieving sustainable development. The evolutionary effect of natural resources on the emissions of CO_2_ is not an inverted U-shape. Currently, natural resources are environmentally friendly but in future due to mismanagement resources will also contaminate the environmental quality by increasing CO_2_ emissions. This result has also highlighted the policy instruments to preserve and use natural resources sustainably. The abundance of natural resources helps to reduce greenhouse gases and also serves as a catalyst for sustainable growth. Therefore, nations should be aware of the need to conserve natural resources. 

This work validates the existence of EKC between globalization, GDP, and environmental quality. This means that globalization is currently creating environmental problems but, in the future, will start to improve air quality by reducing CO_2_ emissions. This finding sheds light on the importance of globalization for the G-11 nations. It is expected that the G-11 nations should explore more markets to export their products, especially to the developed nations. This will provide the nations with the opportunities to import cleaner technologies to deal with CO_2_ emissions. 

Apart from the contribution of this study, future research can be applied to highly globalized and developed countries. Moreover, future works can be undertaken by utilizing the other panel data analysis. 

## Figures and Tables

**Table 1:** Descriptive statistics.

Parameters	Mean	Minimum	Maximum	Standard Deviation	Skewness	Kurtosis
**CO_2_e**	57,553	2020	583,110	108,944.2	2.97	8.72
**GDP**	100,953,498,375.5	4,689,605,208.6	1,049,318,966,508.5	176,187,954,357.7	3.29	11.55
**GL**	58.47	32.23	80.89	10.28	−0.10	−0.26
**NAT**	2.44	0.03	18.85	3.44	2.50	6.65
**AG**	2,220,265.9	113,959	17,129,349	3,495,883.2	2.18	4.0

**Table 2:** Description of the parameters under study.

Parameters	Symbol	Unit	Source
Carbon Dioxide emissions	CO_2_	kilo ton (kt)	World Bank
Globalization	GL	Overall Index (Economic, political, and social globalization)	KOF institute
Gross Domestic Product	GDP	Constant 2015 US$	World Bank
Natural Resource abundance	NAT	Natural resource rents (%GDP)	World Bank
Research and Development	RD	Number of patents (residents)	World Bank
Ageing population	AG	Population more than 65 years	World Bank

**Table 3:** Results obtained for cross-section dependence (CD) analysis.

Variable	Test Statistics (*p*-Values)
CO_2_	20.45 *** (0.00)
GL	16.76 *** (0.00)
GDP	19.65 *** (0.00)
NAT	44.23 *** (0.00)
AG	27.67 *** (0.00)
RD	32.34 *** (0.00)

*** is significant at 1%.

**Table 4:** CIPS unit root test results from the study.

Variable	CIPS Test	
	At Level	1st Difference
CO_2_	−2.94	−5.61 ***
GL	−2.78	−5.86 ***
GDP	−2.65	−6.90 ***
NAT	−3.52 ***	−6.16 ***
AG	−1.01	−3.45 **
RD	−3.12 ***	−6.10 ***

** and *** are significant at 5% and 1% levels, respectively.

**Table 5:** Results obtained to show the slope heterogeneity.

Statistics	Test Value (*p*-Value)
Delta-tilde	23.46 *** (0.00)
Delta-tilde Adjusted	26.57 *** (0.00)

*** is significant at 1% level.

**Table 6:** Westerlund and Edgerton [53] results obtained for panel co-integration test.

Test	No Shift	Mean Shift	Regime Shift
Z_φ_(N)	−3.56 ***	−2.87 ***	−4.02 ***
P_value_	0	0	0
Z_τ_(N)	−4.67 ***	−3.67 ***	−4.01 ***
P_value_	0	0	0

*** is significant at 1% level

**Table 7:** Cross-sectional autoregressive distributed lag (CS-ARDL) results from the study.

Short Run	Coefficient	Std. Error	Significance Level
ΔCO_2_	−0.95 ***	0.09	0.00
ΔGL	0.24 **	0.25	0.05
ΔGL^2^	−2.32	2.24	0.78
ΔGDP	9.75 **	3.84	0.01
ΔGDP^2^	−1.60 ***	0.70	0.02
ΔNAT	−0.05 ***	0.02	0.09
ΔNAT^2^	0.02	0.03	0.53
ΔAG	−23.20 ***	25.40	0.36
ΔAG^2^	1.60	1.84	0.35
ΔRD	−0.07 **	0.01	0.53
ΔEN	2.32 ***	0.87	0.00
Long Run			
CO_2_	−0.04 **	0.05	0.09
GL	0.09	0.13	0.09
GL^2^	−2.32 **	2.19	0.03
GDP	5.24 ***	2.11	0.00
GDP^2^	−0.84 **	0.38	0.06
NAT	−0.07	0.01	0.53
NAT^2^	0.01	0.02	0.54
AG	−13.41**	13.48	0.03
AG^2^	0.91	0.99	0.02
RD	−0.46 ***	0.06	0.03
EN	1.65 **	0.74	0.01
ECT	−0.95 ***	0.09	0.00

** and *** are significant at 5% and 1% levels, respectively.

## Data Availability

Sources of data used are provided in the text.
